# Rethinking the course of psychotic disorders: modelling long-term symptom trajectories

**DOI:** 10.1017/S0033291720004705

**Published:** 2022-10

**Authors:** Craig Morgan, Paola Dazzan, Julia Lappin, Margaret Heslin, Kim Donoghue, Paul Fearon, Peter B Jones, Robin M Murray, Gillian A Doody, Ulrich Reininghaus

**Affiliations:** 1 ESRC Centre for Society and Mental Health, Institute of Psychiatry, Psychology, and Neuroscience, King's College, London, UK; 2 National Institute for Health Research (NIHR) Mental Health Biomedical Research Centre at South London and Maudsley NHS Foundation Trust and King's College London, London, UK; 3 Department of Psychological Medicine, Institute of Psychiatry, Psychology, and Neuroscience, King's College, London, UK; 4 Faculty of Medicine, University of New South Wales, Sydney, Australia; 5 King's Health Economics, Health Service and Population Research Department, Institute of Psychiatry, Psychology, and Neuroscience, King's College, London, UK; 6 Addictions Department, Institute of Psychiatry, Psychology, and Neuroscience, King's College, London, UK; 7 Department of Psychiatry, Trinity College, Dublin, Ireland; 8 Department of Psychiatry, University of Cambridge, Cambridge, UK; 9 Psychosis Studies Department, Institute of Psychiatry, Psychology, and Neuroscience, King's College, London, UK; 10 Division of Psychiatry and Applied Psychology, University of Nottingham, Nottingham, UK; 11 Department of Public Mental Health, Central Institute of Mental Health, Medical Faculty Mannheim, University of Heidelberg, Mannheim, Germany

**Keywords:** Psychotic disorder, course and outcome, epidemiology, growth mixture models

## Abstract

**Background:**

The clinical course of psychotic disorders is highly variable. Typically, researchers have captured different course types using broad pre-defined categories. However, whether these adequately capture symptom trajectories of psychotic disorders has not been fully assessed. Using data from AESOP-10, we sought to identify classes of individuals with specific symptom trajectories over a 10-year follow-up using a data-driven approach.

**Method:**

AESOP-10 is a follow-up, at 10 years, of 532 incident cases with a first episode of psychosis initially identified in south-east London and Nottingham, UK. Using extensive information on fluctuations in the presence of psychotic symptoms, we fitted growth mixture models to identify latent trajectory classes that accounted for heterogeneity in the patterns of change in psychotic symptoms over time.

**Results:**

We had sufficient data on psychotic symptoms during the follow-up on 326 incident patients. A four-class quadratic growth mixture model identified four trajectories of psychotic symptoms: (1) remitting-improving (58.5%); (2) late decline (5.6%); (3) late improvement (5.4%); (4) persistent (30.6%). A persistent trajectory, compared with remitting-improving, was associated with gender (more men), black Caribbean ethnicity, low baseline education and high disadvantage, low premorbid IQ, a baseline diagnosis of non-affective psychosis and long DUP. Numbers were small, but there were indications that those with a late decline trajectory more closely resembled those with a persistent trajectory.

**Conclusion:**

Our current approach to categorising the course of psychotic disorders may misclassify patients. This may confound efforts to elucidate the predictors of long-term course and related biomarkers.

## Introduction

Our knowledge of the nature and predictors of the long-term course and outcomes of psychotic disorders is surprisingly limited. In a previous attempt to synthesise the evidence, we identified only 13 studies, published up to 2014, that had followed individuals with a first episode of psychosis and reported on course and outcome over a period of 8 years or more (Morgan et al., 2014). We are aware of two further studies since then (Kotov et al., 2017; Secher et al., 2015). This body of research is methodologically varied, which makes direct comparisons difficult and limits any general conclusions we can make about the long-term trajectories of psychosis following a first episode and associated factors.

Further, our characterisation of the symptomatic course of psychotic disorders is mostly crude and static. Following Ciompi (1980), researchers have tended to characterise symptomatic course by assigning individuals to broad groups based on lengths of episodes and remissions (Andreasen et al., 2005; Harrison et al., 2001; Hopper, Harrison, Janca, & Sartorius, 2007; Moller et al., 2010; White et al., 2009). For example, in one common characterisation, an episodic course is defined as one in which no episode of psychosis lasted 6 months or more; a continuous course as one in which no remission lasted 6 months or more; and neither, by default, as one in which there was an episode of 6 months or more and a remission of 6 months or more (e.g. Hopper et al., 2007). The validity of this approach has not, as far as we are aware, been assessed and, by using a pre-defined cut point of 6 months to define episodes and remissions, it may misclassify individuals with similar overall trajectories and consequently confound efforts to identify the predictors of course and outcome.

However, developing more fine-grained approaches to investigating the long-term course of psychotic disorders presents several methodological challenges. Even categorising course as above requires reconstructing periods of relapse and remission over many years. This said, the use of multiple sources and informants, life course interview techniques and consensus ratings can improve the validity of retrospective reconstructions of periods of symptoms (Bebbington et al., 2006). However, analysing detailed data on fluctuations in symptoms over many years presents further challenges and this probably explains why these fluctuations are usually distilled into a small number of static categories. There are statistical methods that offer an alternative, by enabling a data-driven approach to identifying individuals whose symptoms follow similar trajectories over time (Austin et al., 2015; Muthen, 2004; Muthen & Muthen, 2000). For example, latent growth mixture models can identify trajectories (i.e. categorical latent variables) that account for heterogeneity in fluctuations of psychotic symptoms over time (Grimm, Ram, & Estabrook, 2017). Such an approach may produce more valid groupings and enable us to better elucidate the predictors of course and outcome.

Using extensive data from AESOP-10, collated from multiple sources and meticulously rated by consensus, we sought to examine fluctuations in symptoms over a 10-year follow-up period. Specifically, we sought to identify classes of individuals with specific symptom trajectories over a 10-year follow-up and to, then, compare trajectories with usual categories of course and examine associations between trajectories and baseline demographic, social and clinical characteristics.

## Method

AESOP-10 is a follow-up at approximately 10 years of a cohort of 532 incident cases with a first episode of non-affective or affective psychosis initially identified in South East London and Nottingham, UK. There were marked differences in incidence between the two sites [i.e. adj. incidence rate 49.4 (95% CI 43.6–55.3) per 100 000 person-years in London and 23.9 (95% CI 20.6–27.2) per 100 000 in Nottingham; see Kirkbride et al., 2006].

### Ethics

Ethical approval was provided by the Joint South London and Maudsley and Institute of Psychiatry NHS Research Ethics Committee (Ref: 321/02) and the North Nottinghamshire Local Research Ethics Committee (Ref: 04/Q2402/35). The authors assert that all procedures contributing to this work comply with the ethical standards of the relevant national and institutional committees on human experimentation and with the Helsinki Declaration of 1975, as revised in 2008.

### Follow-up procedures

At baseline, detailed re-contact information was collected for all patients. At approximately 10 years, we sought to re-contact and re-interview each patient. All deaths and emigrations up to 12 December 2012 were identified through the Office for National Statistics (ONS) for England and Wales and the General Register Office (GRO) for Scotland [full details on mortality in the AESOP-10 cohort can be found in Reininghaus et al. (2015)].

### Data (1) baseline

At baseline, all individuals with a first-episode psychotic disorder who presented to secondary mental health services in our catchment areas over 2 years were identified from 1997 to 1999 (i.e. before early intervention services were introduced in the UK). This predates the introduction of early intervention services in the UK and all consequently received standard inpatient and community care. Data were collected on clinical presentation, sociodemographic characteristics, and a range of neurodevelopmental and social risk factors. We estimated premorbid Intelligence Quotient (IQ) using the National Adult Reading Test (NART; Nelson, 1991). To capture exposure to multiple disadvantages and isolation, we constructed an index by counting the presence of the following: unemployment, living alone, living in rented housing and being single.

### Data (2) follow-up

At follow-up, information was collated across three domains (clinical, social and service use) using an extended version of the WHO Life Chart (Susser et al., 2000) and the Global Assessment of Function Disability (GAF-D) Scale (Endicott, Spitzer, Fleiss, & Cohen, 1976). We extended the WHO Life Chart to include a timeline to record the presence or absence of psychotic symptoms month by month during the follow-up period. Information on psychotic symptoms was collected from interviews with patients and, with consent, from informants (e.g. clinicians, relatives) and case records (Morgan et al., 2014). Interviews used life course techniques, including anchoring around key events (e.g. birthdays, hospital admissions, etc.), to maximise recall. Using all information from follow-up interviews with patients and treating clinicians (informants) and from clinical records, researchers methodically reconstructed patient histories over the follow-up period. Ratings of the presence or absence of psychotic symptoms were based on Schedules for Clinical Assessment in Neuropsychiatry (SCAN) (WHO, 1992) criteria for rating of clinically important symptoms and were only made on the basis of clear information indicating presence or absence (i.e. clear description of the occurrence of symptoms during a specified period at a sufficient level of intensity to be rated present according to SCAN criteria); otherwise, data were coded as missing. All final ratings of presence, absence or missing (i.e. insufficient information to make a rating) were made by consensus at weekly meetings involving members of the research team and a senior psychiatrist. In addition, information on substance abuse and dependence and markers of social disadvantage and isolation across a number of domains during and at follow-up was collected using the Life Chart. The GAF-D Scale, which provides information on overall social function, was completed for the 1-month pre-follow-up.

### Analysis

Growth mixture modelling was used to identify latent trajectory classes (i.e. categorical latent variables) that account for heterogeneity in the patterns of change in psychotic symptoms over time (Grimm et al., 2017; Muthen, 2004; Muthen & Aspharouhov, 2008). We fitted a series of models to determine the classification that best represented temporal patterns of change in psychotic symptoms. First, we fitted growth mixture models (GMM) without random intercept and slope (Muthen, 2004; Nagin, 2005). We then added quadratic and cubic terms of time to account for potential non-linear growth in the data. We also examined whether adding random intercepts and slopes that account for heterogeneity in symptom change within latent trajectory classes further improved model fit. Data were assumed to be missing at random (MAR), which allowed for computing unbiased estimates based on all available data using maximum likelihood estimation with robust standard errors (i.e. the MLR estimation method in MPlus). To assess the assumption of MAR, we examined the association between socio-demographic and clinical characteristics at baseline and missingness in timeline data over 10 years of follow-up fitting random-intercept logistic regression models. GMM fit was assessed using the Bayesian Information Criterion (BIC) (Schwarz, 1978), with lower values and differences equal to or above 10 indicating better model fit than for the comparison model (Raftery, 1995). We computed the entropy for models with more than one class to examine the quality of classification accuracy. Entropy values closer to 1 indicate a better distinction between latent classes (range 0–1). We also computed average latent class probabilities for most likely latent class membership as another indicator of goodness of model fit or uncertainty in assigning individuals to latent classes given the observed data (Geiser, 2013). GMM were further compared using the Lo–Mendell–Rubin likelihood ratio test (Lo, Mendell, & Rubin, 2001) and a bootstrapped likelihood ratio test with 500 draws (Nylund, Aspharouhov, & Muthen, 2007) to examine whether models with additional classes improved model fit (as indicated by *p* < 0.05), compared with a model with one less class. In determining the number of classes in the final model, we also considered interpretability and class sizes (Uher et al., 2010). Specifically, we only included additional latent classes if they represented fundamentally different trajectories rather than minor variations of those included in the previous model (Muthen, 2004). Further, we aimed for a final model without latent class trajectories including only a very small proportion of subjects (<5%). Trajectories including equal to or more than 5% of subjects were still considered as clinically important, given that identifying and targeting predictors of trajectories of symptom change even in relatively small subpopulations can lead to potentially substantial public health benefits. Analyses were conducted using STATA Version 12 and MPlus Version 7.1. (Muthen & Muthen, 2013).

## Results

### Sample

At baseline, we identified 532 incident cases, of whom 390 consented to complete assessments. At follow-up, of the 532 incident cases, 37 had died, 29 had emigrated and 8 were excluded. Of the remaining 458, we were able to complete a timeline of psychotic symptoms throughout the follow-up for 326 (71% of 458), with a mean length of follow-up of 10.6 years (SD 1.1). Characteristics of the included sample (*n* = 326) *v*. those not included (*n* = 206) are shown in online Supplementary Table S1.

### Missing data

There was no evidence of an association between missingness and age, gender, ethnicity, level of education, social disadvantage, pre-morbid IQ, diagnosis, DUP and mode of onset, and only weak evidence of an association between duration of untreated psychosis and missingness in timeline data over the 10-year follow-up (see online Supplementary Table S2). This suggests the assumption that symptom data are MAR is acceptable.

### Trajectories of symptom change in psychotic symptoms

Overall, GMM with random intercept and slope (online Supplementary Table S4) showed a better model fit than GMM without random intercepts and slopes (online Supplementary Table S3). GMM with linear effects provided a good model fit (online Supplementary Table S4, Models 1.1.1–1.1.6). However, estimated means of linear time effects in these models differed markedly from observed data and model fit was further improved when adding quadratic effects of time to account for non-linear growth in the observed data, with high-quality classification of subjects into latent trajectory classes (entropy >0.90). A lower BIC value was found for GMM with quadratic effects (online Supplementary Table S4, Models 1.2.1–1.2.6) compared with GMM with linear effects (online Supplementary Table S4, Models 1.1.1–1.1.6), whereas GMM with cubic effects yielded a non-positive definite residual covariance matrix and first-order derivative product matrix even after increasing starting values and, hence, pointed to model non-identification.

**Fig. 1.**
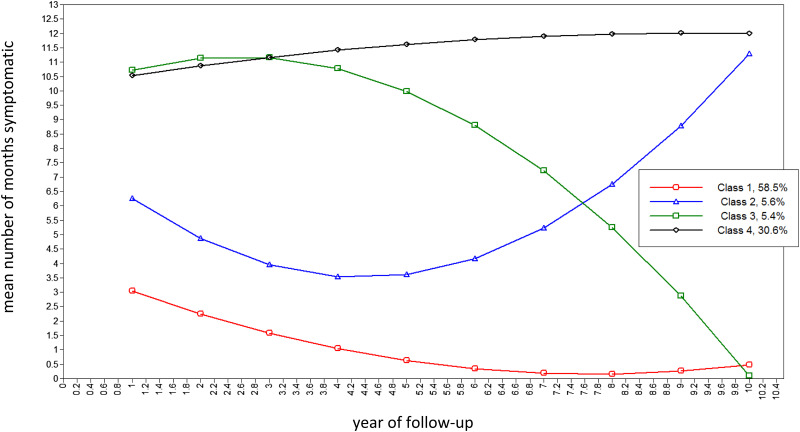
Estimated latent trajectories of 4-class quadratic GMM (Model 2.2.4, see Table 2) for number of months psychotic per year (*n* = 326). Note: Class 1: Remitting: course characterised by remitting periods of symptoms, which became shorter and less frequent over time. Class 2: Late decline: course characterised, initially, by remitting periods of symptoms, with more persistent symptoms over time. Class 3: Late improvement: course characterised, initially, by persistent symptoms, with remitting periods of symptoms later. Class 4: Persistent: a course characterised by persistent or long periods of symptoms throughout.

**Fig. 2.**
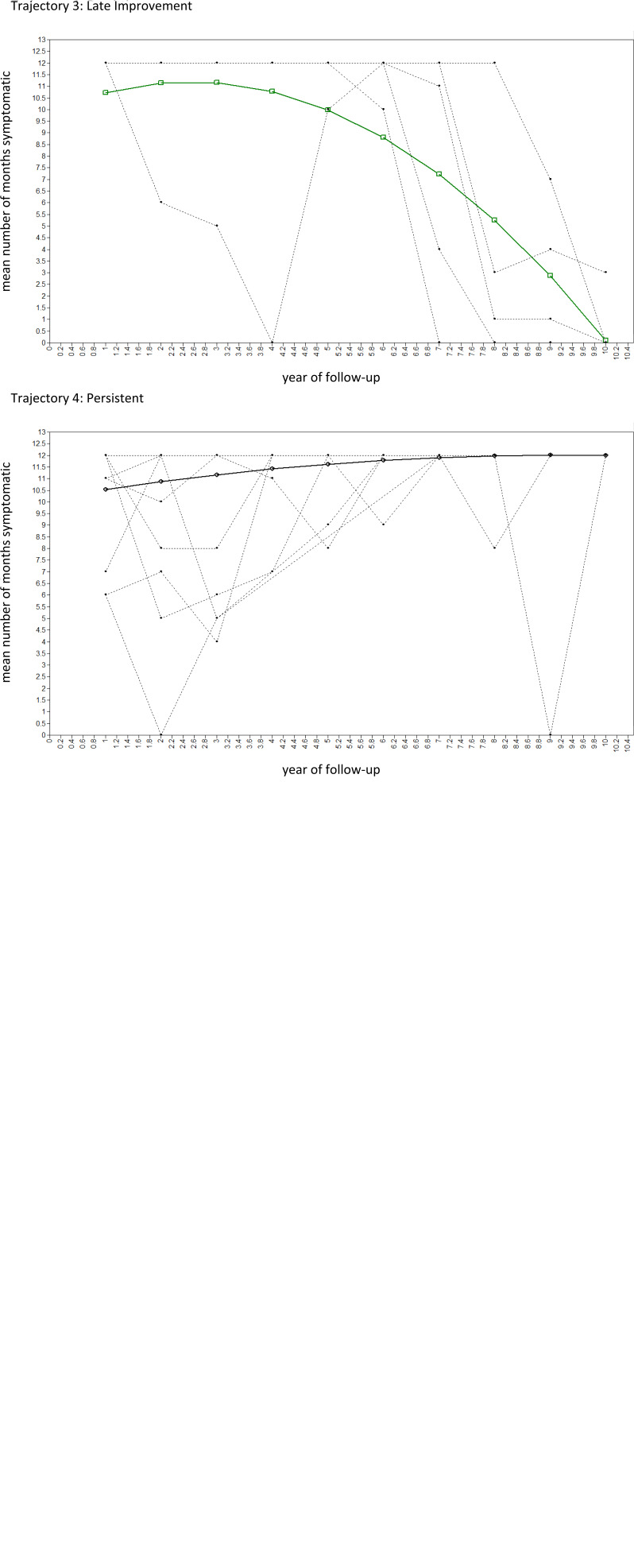
Estimated means and observed values of 4-class quadratic GMM in randomly selected 100 subjects (Model 2.2.4, see Table 2) for number of months psychotic per year (*n* = 326).

The BIC and Lo–Mendell–Rubin test indicated a markedly improved model fit for the quadratic GMM with two latent trajectory classes (online Supplementary Table S4; online Supplementary Figs S1 and S2). However, there was still marked departure of observed temporal patterns of changes in psychotic symptoms from estimated means in this quadratic, two-class GMM (online Supplementary Table S5). In line with this, the BIC and bootstrapped likelihood ratio test indicated that model fit continued to improve for the 3-, 4-, 5- and 6-class quadratic GMM (online Supplementary Table S4). However, there was a lower entropy for the 5- and 6-class quadratic GMM compared with the 4-class quadratic GMM. Given also that the 5- and 6-class quadratic GMM included trajectories with <5% of subjects, the 4-class quadratic GMM was selected as the final, best-fitting model (see Figs 1 and 2; online Supplementary Tables S4 and S6), in which average latent class probabilities for most likely class membership were very high (i.e. >0.98; see online Supplementary Table S6). The classes can be characterised as remitting-improving (Class 1, 58.5%), late decline (Class 2, 5.6%), late improvement (Class 3, 5.4%) and persistent (Class 4, 30.6%) trajectories.

### Latent trajectories and other course and outcome variables

When compared with the usual classification of course types (episodic, continuous and neither), the two largest classes captured all those in the episodic (*n* = 94 of 94; 100%) and almost all those in the continuous (*n* = 72 of 78; 92%) categories. A majority of those in neither category were classified in the remitting class (i.e. *n* = 90 of 145; 62%), with the remainder spread across the other three classes (i.e. *n* = 25, 17% persistent; *n* = 14, 10% late decline; *n* = 16, 11% late improvement) (Table 1). With regard to symptom recovery (i.e. symptom free for the preceding 2 years), almost three-quarters (73%; 124 of 179) of those in the remitting class had recovered symptomatically at follow-up. By definition, none of those in the continuous class or the late decline class had recovered. Among the small class of individuals whose symptoms improved late, more than half (56%; 9 of 17) had recovered at follow-up.

**Table 1.** Latent trajectories and other clinical course and outcome variables

Trajectories	No further episode^a^ *n* (%)		Episodic^a^ *n* (%)		Neither^a^ *n* (%)		Continuous^a^ *n* (%)		Recovered^b^ *n* (%)		Not recovered^b^ *n* (%)	
1 Remitting	34	(100.0)	60	(100.0)	90	(62.1)	3	(3.9)	124	(93.2)	45	(28.5)
2 Late decline	–	–	–	–	16	(11.0)	1	(1.3)	–	–	16	(10.1)
3 Late improvement	–	–	–	–	14	(9.7)	2	(2.6)	9	(6.8)	7	(4.4)
4 Persistent	–	–	–	–	25	(17.2)	72	(92.3)	–	–	90	(57.0)

Note 1: Class 1: Remitting: course characterised by remitting periods of symptoms, which became shorter and less frequent over time; Class 2: Late decline: course characterised, initially, by remitting periods of symptoms, with more persistent symptoms over time; Class 3: Late improvement: course characterised, initially, by persistent symptoms, with remitting periods of symptoms later; Class 4: Persistent: a course characterised by persistent or long periods of symptoms throughout.

Note 2: Given that many cells have 0 or a small number of observations, and patterns of overlap between trajectories and other course and outcome variables are clear, test statistics were not calculated.

a Missing, *n* = 9.

b Missing, *n* = 35.

Further, there were strong associations between latent symptom trajectories and social outcomes (Table 2). For example, the mean GAF disability score at follow-up was almost 20 points higher among those in the remitting class compared with those in the persistent class [mean GAF-D: remitting 63.7 (s.d. 17.9) *v*. persistent 45.9 (s.d. 12.2); *F*_30.0_, df 3, *p* < 0.001]. The mean scores for the late decline (41.2; s.d. 17.4) and improvement (49.8; s.d. 12.4) classes were similar to that for the persistent group (45.9; s.d. 12.2). On broad markers of social exclusion, over 60% of those in the remitting class were not in a long-term relationship at follow-up and over 60% were unemployed. These proportions were, not surprisingly, lower still among those in the persistent class, particularly for those employed (<10%). Even so, around 20% who were more or less continuously symptomatic were in a relationship, which underscores the importance of considering symptomatic and social outcomes separately.

**Table 2.** Latent trajectories and social course and outcome variables

Trajectories	In employment^a^ row *n* (row %)		Not in employment^a^ row *n* (row %)		In a relationship^b^ row *n* (row %)		Not in a relationship^b^ row *n* (row %)		GAF-D Mean (SD)	
1 Remitting	49	(32.0)	104	(68.0)	59	(37.3)	99	(62.7)	63.7	(17.9)
2 Late decline	1	(7.1)	13	(92.9)	5	(31.3)	11	(68.8)	41.2	(17.4)
3 Late improvement	1	(6.7)	14	(93.3)	3	(23.1)	10	(76.9)	49.8	(12.4)
4 Persistent	7	(7.4)	88	(92.6)	17	(18.1)	77	(81.9)	45.9	(12.2)
Test statistics	χ^2^ 25.38; df 3; *p* < 0.001				χ^2^ 10.74; df 3; *p* = 0.013				*F*30.0; df 3; *p* < 0.001	

Note: Class 1: Remitting: course characterised by remitting periods of symptoms, which became shorter and less frequent over time; Class 2: Late decline: course characterised, initially, by remitting periods of symptoms, with more persistent symptoms over time; Class 3: Late improvement: course characterised, initially, by persistent symptoms, with remitting periods of symptoms later; Class 4: Persistent: a course characterised by persistent or long periods of symptoms throughout.

^a^Missing, *n* = 49.

^b^Missing, *n* = 45.

### Demographic, social and clinical characteristics

There were several strong associations between baseline demographic, social and clinical variables, which closely mirror previous findings (Table 3 and online Supplementary Table S7). For example, compared with the remitting class, in the persistent class, there were more men (Adj. OR 1.89, 95% CI 1.13–3.17), more from London (Adj. OR 1.58, 95% CI 0.84–2.96), more of black Caribbean ethnicity (*v*. white British, Adj. OR 1.96, 95% CI 1.01–3.80), more with lower levels of education (e.g. school *v*. university, Adj. OR 3.86, 95% CI 1.20–12.39) and with higher levels of social disadvantage and isolation (e.g. 4 markers *v*. 0 or 1, Adj. OR 2.72, 95% CI 1.08–6.88), more in the lowest quantile of premorbid IQ [e.g. lowest *v*. highest quartile, Adj. OR 3.35 (0.82–13.50)], fewer with a diagnosis of an affective psychotic disorder (Adj. OR 0.17, 95% CI 0.08–0.38), and DUP was substantially longer (Adj. OR for each additional month DUP 1.05, 95% CI 1.01–1.09). Not surprisingly, in the persistent class, there were more who were treatment-resistant from the outset [*n* = 36 of 46 (78%); online Supplementary Table S8]. There was no strong evidence of differences in substance abuse or dependence among those in the persistent *v*. remitting class.

**Table 3.** Baseline socio-demographic and clinical characteristics by latent trajectories, odds ratios

	Late decline *v*. remitting		Late improvement *v*. remitting OR (95% CI)		Persistent *v*. remitting OR (95% CI)	
	Unadj. OR (95% CI)	Adj. OR^a^ (95% CI)	Unadj. OR (95% CI)	Adj. OR^a^ (95% CI)	Unadj. OR (95% CI)	Adj. OR^a^ (95% CI)
Study centre						
London *v*. Nottingham	1.12 (0.40–3.17)	0.55 (0.16–1.91)	1.84 (0.57–5.92)	2.09 (0.54–8.11)	1.78 (1.04–3.01)	1.58 (0.84–2.96)
Sex						
Men *v*. women	0.85 (0.32–2.30)	0.88 ((0.32–2.46)	2.11 (0.71–6.30)	2.26 (0.74–6.94)	1.73 (1.05–2.84)	1.89 (1.13–3.17)
Age	0.99 (0.94–1.05)	0.99 (0.94–1.05)	0.99 (0.93–1.04)	0.98 (0.93–1.04)	1.00 (0.99–1.03)	1.01 (0.99–1.04)
Ethnicity						
Other White *v*. White British	3.33 (0.55–20.22)	4.44 (0.66–29.60)	0.95 (0.11–8.43)	0.62 (0.06–5.98)	1.72 (0.64–4.61)	1.28 (0.45–3.62)
Black Caribbean *v*. White British	2.98 (0.83–10.72)	3.89 (0.92–16.52)	1.46 (0.46–4.60)	1.14 (0.32–4.10)	2.25 (1.25–4.06)	1.96 (1.01–3.80)
Black African *v*. White British	2.31 (0.49–11.00)	3.43 (0.55–21.51)	0.88 (0.17–4.50)	0.59 (0.10–3.48)	1.09 (0.48–2.47)	0.89 (0.36–2.19)
Education						
Further *v*. university	3.48 (0.40–30.35)	2.65 (0.28–24.95)	2.32 (0.25–21.76)	1.84 (0.18–18.67)	4.50 (1.44–14.02)	3.67 (1.13–11.89)
GCSE *v*. university	2.84 (0.32–25.53)	2.45 (0.25–23.79)	1.37 (0.10–13.09)	0.82 (0.07–10.33)	3.70 (1.17–11.64)	2.95 (0.90–9.63)
School *v*. university	2.15 (0.23–20.12)	1.55 (0.15–15.72)	4.83 (0.58–40.05)	4.07 (0.45–36.72)	4.83 (1.57–14.92)	3.86 (1.20–12.39)
Social disadvantage						
2 *v*. 0, 1	0.52 (0.05–5.99)	0.43 (0.03–5.20)	1.57 (0.25–9.88)	1.29 (0.19–8.66)	2.51 (1.07–5.88)	2.11 (0.87–5.14)
3 *v*. 0, 1	2.68 (0.49–14.60)	2.08 (0.33–12.97)	1.07 (0.14–7.97)	0.93 (0.11–7.76)	2.47 (1.05–5.81)	2.11 (0.85–5.25)
4 *v*. 0, 1	3.93 (0.71–21.65)	3.66 (0.60–22.36)	4.17 (0.89–25.02)	4.92 (0.84–28.70)	3.46 (1.43–8.38)	2.72 (1.08–6.88)
Substance use						
Abuse *v*. non-problematic use	0.46 (0.10–2.17)	0.51 (0.10–2.68)	2.08 (0.44–9.76)	2.05 (0.38–11.14)	1.45 (0.78–2.67)	1.40 (0.70–2.82)
Depend. *v*. non-problematic use	0.21 (0.03–1.66)	0.19 (0.02–1.67)	5.63 (1.64–19.31)	5.31 (1.20–23.49)	1.04 (0.55–1.97)	0.95 (0.45–1.99)
Diagnosis						
Non-affective *v*. affective	0.23 (0.05–1.02)	0.26 (0.06–1.89)	0.77 (0.26–2.32)	1.16 (0.36–3.66)	0.17 (0.08–0.35)	0.17 (0.08–0.38)
DUP (months)^b^	1.02 (0.94–1.11)	1.02 (0.94–1.11)	1.04 (0.98–1.11)	1.03 (0.97–1.10)	1.06 (1.02–1.10)	1.05 (1.01–1.09)
Premorbid IQ (quartiles)						
2nd *v*. 1st (highest)	–	–	–	–	3.55 (0.97–13.03)	2.42 (0.59–9.91)
3rd *v*. 1st (highest)	–	–	–	–	5.33 (1.53–18.59)	4.81 (1.23–18.87)
4th *v*. 1st (highest)	–	–	–	–	5.06 (1.39–18.38)	3.35 (0.82–13.60)

Note 1: Class 1: Remitting: course characterised by remitting periods of symptoms, which became shorter and less frequent over time; Class 2: Late decline: course characterised, initially, by remitting periods of symptoms, with more persistent symptoms over time; Class 3: Late improvement: course characterised, initially, by persistent symptoms, with remitting periods of symptoms later; Class 4: Persistent: a course characterised by persistent or long periods of symptoms throughout.

Note 2: See online Supplementary Table S6 for frequencies and percentages for each trajectory class by socio-demographic and clinical characteristics.

a Adjusted, as appropriate, for centre, sex, age and ethnicity.

b OR is the increase in odds of a late decline, late improvement, or persistent course (*v*. remitting) trajectory for every additional month of DUP.

In the other two classes – late improvement and decline – the numbers mean it is difficult to discern any clear patterns. Nonetheless, two observations are worth noting. First, those in the late improvement class (i.e. those who gradually improve from around 3 years) had a much shorter DUP, on average, and were more likely to meet the criteria for substance abuse or dependence at some point than those in the persistent class. Second, there were some indications that those with a late decline trajectory more closely resembled those with a persistent trajectory (i.e. more of black Caribbean ethnicity, more at higher levels of social disadvantage, fewer with a diagnosis of affective psychosis and longer DUP compared with remitting) than did those with a late improvement trajectory (i.e. no evidence of demographic and diagnostic differences compared with remitting). Still, given the small numbers, this reading of the data is somewhat speculative and needs to be considered with caution, as the basis for hypotheses to be tested in larger samples.

## Discussion

We sought to use a data-driven approach to identify classes of change in psychotic symptoms over time. Symptom course was highly variable. This noted, we identified four latent trajectory classes. Two were common: i.e. a trajectory characterised by remitting periods of symptoms, which became shorter and less frequent over time, leading to recovery for many (~60%), and a trajectory characterised by persistent or long periods of symptoms over the whole follow-up (~30%), with many meeting treatment-resistant criteria. For most, the pattern of course changes – and improves – over time, a finding in line with the only other attempt we are aware of to use latent class models to capture course types (albeit, this study did not model growth – i.e. change – over time) (Austin et al., 2015). Overall, this suggests slightly more positive symptom outcomes than some studies (e.g. International Study of Schizophrenia, ~50% recovered at 15-year follow-up (Harrison et al., 2001)) and substantially more positive outcomes than in some others (e.g. Suffolk County Study, ~25% with non-continuous illness at 20-year follow-up (Kotov et al., 2017)). That patterns of symptoms do change over time is further highlighted by the other two classes: late decline (~5%) and late improvement (~5%). Further, our analyses identified several baseline characteristics that were associated with a persistent course, i.e. men, black Caribbean ethnicity, urban context (i.e. London), social disadvantage, low premorbid IQ, long DUP and non-affective psychoses.

## Methodological considerations

Our findings need to be considered in light of several methodological limitations. In attempting to retrospectively reconstruct fluctuations in symptoms over a 10-year period, measurement error and missing data are inevitable. We used a number of approaches to minimise measurement error and maximise the reliability of data, including the use of life course interview techniques, multiple sources and informants, and consensus ratings, and only made ratings based on positive evidence of the presence or absence of symptoms. However, we were reliant on recall and use of clinical records, which varied in detail and periods covered. This further increased the amount of missing data. This is relevant at two levels. First, a proportion of patients for whom we did not have sufficient data over the follow-up period could not be included in our analyses. Those with sufficient data and included in analyses were more likely to be from London (*v*. Nottingham) and less likely to be white British. This may have introduced bias. It is possible, for example, that those who were not included may have had better outcomes, on the basis that they had fewer contacts with mental health services, which would bias our findings towards poor outcomes. Conversely, it is possible that those with negative symptoms had fewer contacts with services, which would bias our findings towards better outcomes. Second, data on the presence or absence of symptoms did not include broader measures of outcome such as social functioning, which may have yielded different trajectories as, for example, those identified by Velthorst et al. (2017). Further, WHO Life Chart data were collected for the 10-year follow-up period (i.e. first presentation to follow-up) and not for the period before baseline (i.e. symptom onset to first presentation). Consequently, we cannot rule out that some individuals may have been misclassified into the late decline or persistent trajectories because symptom data were not available for the period before first presentation. Third, of the patients included in the sample, not all had complete data for the entire follow-up period. As indicated already, with the possible exception of DUP, there was no evidence of associations between missingness in the timeline data and sociodemographic and clinical characteristics. From this, we were able to generate unbiased estimates based on all available data, on the assumption that data were MAR. Still, these issues provide important caveats to our data and what can be inferred.

## Rethinking course: implications

These limitations noted, our analyses provide a novel approach to studying the course of disorder and offer potentially important insights.

To begin with, our analyses challenge our way of conceptualising course of psychotic disorders in at least three ways. First, they suggest the use of any pre-specified cut points for remission and relapse to distinguish course types is overly crude and leads to misclassification that may confound efforts to elucidate predictors of course. Second, our findings suggest the use of a middle category of neither, between episodic and continuous, is flawed and may confound findings. This is supported by our previous analyses of MRI data on a sub-sample of AESOP cases and controls using a support vector machine whole-brain classification approach to predict future illness course at the individual level (Mourao-Miranda et al., 2012). Broadly, we found that those in the neither episodic nor continuous group could mostly be categorised according to a discriminating pattern of episodic *v*. continuous. Third, the use of static categories to capture course implies that individuals follow a single trajectory that is unchanging over time. Our data suggest that this is true, at most, for a minority who experience (near) persistent symptoms. For others, the course fluctuates. A majority tend to experience shorter and shorter episodes, a feature that is missed in the simple category of episodic. A small, but noteworthy, proportion experience marked changes in the course of symptoms over time, 3–5 years after a first episode. These groups are important because they suggest an improvement, after an initially poor course, is possible and because they point to a group of individuals who later experience long periods of psychosis, after initial short periods. These findings are likely to be intuitively valid to clinicians who regularly see patients with symptoms that wax and wane over time, and who may disengage from services at times when symptoms are absent. This highlights the need for clinical teams to regularly monitor for psychotic symptoms and the need for fluid access to, and exit from, mental health services, with re-entry to specialist services available at times when symptoms recur.

As noted, we found several broad demographic, social and clinical variables were associated with the course and outcome over the long-term. However, our ability to predict long-term prognosis – particularly at an individual level in clinical practice – is still limited (Suvisaari et al., 2018). Diagnosis provides perhaps the best clinical indicator of prognosis – as we would expect – but there remains considerable heterogeneity in outcomes within diagnostic groups. Our findings hint at several reasons for this. For example, potential issues with measurement error and missing data notwithstanding, our findings highlight the marked variability in fluctuations in symptoms over time. We can discern broad trends, as our identified classes illustrate, and associated factors, but it remains that what lies behind these is considerable heterogeneity in the patterns of remission and relapse over time. This is intrinsically hard to predict. Further, it seems that baseline predictors of outcomes may weaken over time (Dazzan et al., 2020). There is a considerable literature on prognostic markers for short- to medium-term outcomes, including substance use (especially cannabis) (Bozzatello, Bellino, & Rocca, 2019; Schoeler et al., 2016), neurocognition (Kravariti et al., 2019; Lepage, Bodnar, & Bowie, 2014), negative symptoms (Suvisaari et al., 2018) and various biomarkers (e.g. brain structure) (Dazzan et al., 2015; McGuire & Dazzan, 2017). However, findings are often inconsistent and it is unclear to what extent these predict longer-term outcomes. Indeed, putting our findings in this paper together with findings from other analyses of AESOP-10 data, there is a relatively small number of baseline demographic, social and clinical characteristics that are associated with long-term course and outcome and the strongest predictor of later outcomes is early outcomes (Dazzan et al., 2020; Demjaha et al., 2017; Lappin et al., 2014). This hints at a dynamic process in which initial drivers of onset and early course interact with early outcomes to shape subsequent exposure to intervening factors that – over time – are more important in shaping course (e.g. treatment response, substance use, social circumstances, etc.), creating dynamic feedback loops that lead – for most – to idiosyncratic patterns of symptom remission and relapse. At present, we do not have the studies, with sufficient depth of data, to enable more dynamic models to be tested.

Of particular interest from our analyses are those whose trajectories change notably late in the course of illness. Those who experience a late decline appear similar at baseline demographically and clinically to those who experience near persistent symptoms (e.g. longer DUP). The small number in this class urges caution, but if validated in subsequent studies this has potentially important clinical implications and suggests those with, for example, a long DUP may need to be more carefully assessed and treated over time, even if the initial trajectory is positive. Further, those who improve and recover late in the course of their illness may shed light on what can be done to promote recovery when the initial course is poor. There are few clues to this in our data, because the number in this group was small, but it is an important potential avenue for future research. Particular consideration, for example, should be given to the potential impacts on improving later outcomes of clozapine, reduced substance use and greater social inclusion.

## Conclusion

Symptomatic course, following a first-episode psychosis, is heterogeneous and intrinsically difficult to predict, especially over the long-term. This poses considerable challenges for both research and clinical practice. With the advent of electronic clinical records in many services around the world and the development of statistical methods that enable more sophisticated modelling of change over time, illustrated by analyses presented in this paper, there are opportunities for a new wave of research on the course and outcome in psychotic disorders. Of particular importance, there is a substantial sub-group – as ours and other studies highlight – that experiences near persistent symptoms throughout, often despite treatment with antipsychotic medication. The associated suffering, needs and costs are hard to over-state. This underscores the importance of investment both in research and in the continued development of interventions and services to better understand, predict and support those whose symptoms persist over the long-term despite current treatment options.

## Supplementary material

For supplementary material accompanying this paper visit https://doi.org/10.1017/S0033291720004705. click here to view supplementary material
