# Supplementary material for: Rethinking the course of psychotic disorders: modelling long-term symptom trajectories
Source: Psychol Med. 2021 Feb 4;52(13):2641–50. doi: 10.1017/S0033291720004705 (PMC9647538; doi:10.1017/S0033291720004705)
Supplement: Supplementary file 1 [file S0033291720004705sup001.docx]

**Supplementary Tables and Figures**

**Supplementary Table 1.** Demographic and clinical characteristics of sample (included vs. not included).

|  | | Included  n = 326 | | Not included  n = 206 | | df | x^2^, t, z | p |
| --- | --- | --- | --- | --- | --- | --- | --- | --- |
| Centre | |  |  |  |  |  |  |  |
|  | Nottingham | 109 | (33.4) | 94 | (45.6) | 1 | 7.96 | 0.005 |
|  | London | 217 | (66.6) | 112 | (54.4) |  |  |  |
| Sex | |  |  |  |  |  |  |  |
|  | Men | 182 | (55.8) | 128 | (62.1) | 1 | 2.07 | 0.151 |
|  | Women | 144 | (44.2) | 78 | (37.9) |  |  |  |
| Baseline age | |  |  |  |  |  |  |  |
|  | Mean (years) | 29.9 | | 31.9 | | 530 | 2.22 | 0.027 |
|  | sd | 9.9 | | 11.7 | |  |  |  |
| Ethnicity | |  |  |  |  |  |  |  |
|  | White British | 122 | (37.4) | 113 | (54.9) | 5 | 22.20 | <0.001 |
|  | Other White | 23 | (7.1) | 14 | (6.8) |  |  |  |
|  | Black Caribbean | 101 | (31.0) | 34 | (16.5) |  |  |  |
|  | Black African | 42 | (12.9) | 25 | (12.1) |  |  |  |
|  | Asian (all) | 20 | (6.1) | 6 | (2.9) |  |  |  |
|  | Other | 18 | (5.5) | 14 | (6.8) |  |  |  |
| Baseline diagnosis | |  |  |  |  |  |  |  |
|  | Non-affective | 239 | (73.3) | 148 | (71.8) | 1 | 0.14 | 0.711 |
|  | Affective | 87 | (26.7) | 58 | (28.2) |  |  |  |
| DUP* | |  |  |  |  |  |  |  |
|  | Median (weeks) | 8.6 | | 8.1 | | - | -0.09 | 0.926 |
|  | IQR | 2.1-31.9 | | 2.2-41.5 | |  |  |  |

* 23 missing

**Supplementary Table 2.** Associations between baseline sample characteristics and missingness in timeline data.

|  | | Unadj. OR | 95% CI | p |
| --- | --- | --- | --- | --- |
| Age | |  |  |  |
|  | 16-29 | 1.0 |  |  |
|  | 30-65 | 0.66 | 0.37 – 1.19 | 0.167 |
| Sex | |  |  |  |
|  | Men | 1.0 |  |  |
|  | Women | 0.76 | 0.42 – 1.37 | 0.362 |
| Ethnicity | |  |  |  |
|  | White British | 1.0 |  |  |
|  | Other White | 0.40 | 0.11 – 1.37 | 0.142 |
|  | Black Caribbean | 0.70 | 0.35 – 1.41 | 0.321 |
|  | Black African | 0.91 | 0.36 – 2.31 | 0.847 |
|  | Asian (all) | 1.91 | 0.57 – 6.39 | 0.296 |
|  | Other | 0.69 | 0.18 – 2.64 | 0.586 |
| Level of education | |  |  |  |
|  | Other | 1.0 |  |  |
|  | No qualifications | 1.17 | 0.62 – 2.22 | 0.635 |
| Social disadvantage | |  |  |  |
|  | 0, 1 | 1.0 |  |  |
|  | 2 | 1.47 | 0.44 – 4.95 | 0.534 |
|  | 3 | 1.16 | 0.34 – 3.88 | 0.815 |
|  | 4 | 0.49 | 0.14 – 1.69 | 0.258 |
| Premorbid IQ | | 0.98 | 0.95 – 1.02 | 0.317 |
| Baseline diagnosis | |  |  |  |
|  | Non-affective | 1.0 |  |  |
|  | Affective | 1.10 | 0.57 – 2.12 | 0.783 |
| DUP* | | 0.997 | 0.993 to 1.001 | 0.074 |
| Mode of onset | |  |  |  |
|  | Acute | 1.0 |  |  |
|  | Insidious | 0.85 | 0.46 – 1.57 | 0.608 |

* 23 missing

**Supplementary Table 3.** Model fit of Growth Mixture Model (GMM) without random intercept and slopes for number of months psychotic per year (n=326)

| Model | Classes | LL | BIC | Entropy | LMRT | |  | BLRT | |  | Proportion of individuals in class | | | | | |
| --- | --- | --- | --- | --- | --- | --- | --- | --- | --- | --- | --- | --- | --- | --- | --- | --- |
|  |  |  |  |  | 2LL | p |  | 2LL | P |  | 1 | 2 | 3 | 4 | 5 | 6 |
| Linear GMM |  |  |  |  |  |  |  |  |  |  |  |  |  |  |  |  |
| Model 2.1.1 | 1 | -8371.97 | 16813.38 |  |  |  |  |  |  |  | 1.00 |  |  |  |  |  |
| Model 2.1.2 | 2 | -6739.31 | 13565.43 | .99 | 3087.47 | .003 |  |  | <.001 |  | .62 | .38 |  |  |  |  |
| Model 2.1.3 | 3 | -6407.97 | 12920.10 | .98 | 626.60 | .166 |  |  | <.001 |  | .57 | .09 | .34 |  |  |  |
| Model 2.1.4 | 4 | -6220.97 | 12563.47 | .97 | 353.62 | .196 |  |  | <.001 |  | .07 | .29 | .56 | .08 |  |  |
| Model 2.1.5 | 5 | -6116.44 | 12371.76 | .96 | 197.68 | .426 |  |  | <.001 |  | .51 | .08 | .07 | .06 | .28 |  |
| Model 2.1.6 | 6 | -6020.17 | 12196.60 | .96 | 199.84 | .256 |  |  | (<.001) |  | .28 | .02 | .07 | .07 | .51 | .05 |
|  |  |  |  |  |  |  |  |  |  |  |  |  |  |  |  |  |
| Quadratic GMM |  |  |  |  |  |  |  |  |  |  |  |  |  |  |  |  |
| Model 2.2.1 | 1 | -8367.75 | 16810.73 |  |  |  |  |  |  |  | 1.00 |  |  |  |  |  |
| Model 2.2.2 | 2 | -6694.94 | 13488.25 | .99 | 3207.07 | .002 |  |  | <.001 |  | .37 | .63 |  |  |  |  |
| Model 2.2.3 | 3 | -6377.33 | 12876.19 | .98 | 608.90 | .213 |  |  | <.001 |  | .07 | .58 | .34 |  |  |  |
| Model 2.2.4 | 4 | -6187.63 | 12519.92 | .98 | 363.70 | .108 |  |  | <.001 |  | .08 | .28 | .56 | .07 |  |  |
| Model 2.2.5 | 5 | -6075.66 | 12319.13 | .97 | 214.66 | .492 |  |  | <.001 |  | .29 | .53 | .05 | .06 | .06 |  |
| Model 2.2.6 | 6 | -5976.25 | 12143.47 | .96 | 190.33 | .266 |  |  | (<.001) |  | .04 | .06 | .28 | .06 | .48 | .07 |
|  |  |  |  |  |  |  |  |  |  |  |  |  |  |  |  |  |
| Cubic GMM |  |  |  |  |  |  |  |  |  |  |  |  |  |  |  |  |
| Model 2.3.1 | 1 | -8361.12 | 16803.25 |  |  |  |  |  |  |  | 1.000 |  |  |  |  |  |
| Model 2.3.2 | 2 | -6674.01 | 13457.98 | .99 | 3261.48 | .002 |  |  | <.001 |  | .627 | .373 |  |  |  |  |
| Model 2.3.3 | 3 | -6354.17 | 12847.22 | .98 | 618.33 | .539 |  |  | <.001 |  | .57 | .34 | .09 |  |  |  |
| Model 2.3.4 | 4 | -6131.21 | 12430.24 | .98 | 417.26 | .316 |  |  | <.001 |  | .08 | .28 | .57 | .07 |  |  |
| Model 2.3.5 | 5 | -6027.36 | 12251.48 | .96 | 200.75 | .313 |  |  | <.001 |  | .06 | .29 | .07 | .52 | .06 |  |
| Model 2.3.6 | 6 | -5941.42 | 12108.54 | .98 | 189.97 | .175 |  |  | <.001 |  | .55 | .04 | .06 | .03 | .29 | .04 |

LL, Log-Likelihood; BIC, Bayesian Information Criterion; LMRT, Lo-Mendell-Rubin Likelihood Ratio Test; BLRT, Bootstrapped Likelihood Ratio Test

**Supplementary Table 4.** Model fit of Growth Mixture Models (GMM) for number of months psychotic per year (n 326).

| **Model** | **Classes** | **LL** | **BIC** | **Entropy** | **LMRT** | |  | **BLRT** | |  | **Proportion of individuals in class** | | | | | |
| --- | --- | --- | --- | --- | --- | --- | --- | --- | --- | --- | --- | --- | --- | --- | --- | --- |
|  |  |  |  |  | 2LL | p |  | 2LL | P |  | 1 | 2 | 3 | 4 | 5 | 6 |
| **Linear GMM** |  |  |  |  |  |  |  |  |  |  |  |  |  |  |  |  |
| Model 1.1.1 | 1 | -6530.08 | 13146.96 |  |  |  |  |  |  |  | 1.000 |  |  |  |  |  |
| Model 1.1.2 | 2 | -6270.14 | 12644.45 | .97 | 491.56 | .020 |  | 519.87 | <.001 |  | .642 | .358 |  |  |  |  |
| Model 1.1.3 | 3 | -6196.27 | 12514.06 | .96 | 139.70 | .276 |  | 147.75 | <.001 |  | .353 | .606 | .041 |  |  |  |
| Model 1.1.4 | 4 | -6115.33 | 12369.54 | .97 | 145.32 | .198 |  | 153.69 | <.001 |  | .072 | .314 | .049 | .565 |  |  |
| Model 1.1.5 | 5 | -6056.24 | 12268.72 | .96 | 111.75 | .384 |  | 118.18 | <.001 |  | .534 | .046 | .068 | .311 | .041 |  |
| Model 1.1.6^a^ | 6 | -6005.30 | 12184.20 | .97 | 96.34 | .215 |  | 101.88 | <.001 |  | .516 | .041 | .067 | .055 | .049 | .273 |
|  |  |  |  |  |  |  |  |  |  |  |  |  |  |  |  |  |
| **Quadratic GMM** |  |  |  |  |  |  |  |  |  |  |  |  |  |  |  |  |
| Model 1.2.1 | 1 | -6457.11 | 13024.16 |  |  |  |  |  |  |  | 1.000 |  |  |  |  |  |
| **Model 1.2.2** | **2** | **-6173.63** | **12480.35** | **.97** | **543.48** | **.005** |  | **566.96** | **<.001** |  | **.642** | **.358** |  |  |  |  |
| Model 1.2.3 | 3 | -6115.21 | 12386.66 | .98 | 112.00 | .295 |  | 116.84 | <.001 |  | .570 | .075 | .355 |  |  |  |
| **Model 1.2.4** | **4** | **-6009.52** | **12198.43** | **.98** | **159.00** | **.240** |  | **165.87** | **<.001** |  | **.585** | **.056** | **.054** | **.306** |  |  |
| Model 1.2.5 | 5 | -5941.95 | 12086.44 | .96 | 129.54 | .289 |  | 134.76 | <.001 |  | .053 | .548 | .041 | .051 | .306 |  |
| Model 1.2.6^a^ | 6 | -5898.25 | 12022.20 | .96 | 83.77 | .308 |  | 87.39 | <.001 |  | .046 | .052 | .040 | .307 | .512 | .043 |
|  |  |  |  |  |  |  |  |  |  |  |  |  |  |  |  |  |
| **Cubic GMM** |  |  |  |  |  |  |  |  |  |  |  |  |  |  |  |  |
| Model 1.3.1^a^ | 1 | -6387.79 | 12914.46 |  |  |  |  |  |  |  | 1.000 |  |  |  |  |  |

LL, Log-Likelihood; BIC, Bayesian Information Criterion; LMRT, Lo-Mendell-Rubin Likelihood Ratio Test; BLRT, Bootstrapped Likelihood Ratio Test

^a^ Residual covariance matrix and first-order derivative product matrix not positive definite for this and all subsequent models with a higher number of classes

**Supplementary Table 5.** Estimated and observed means of two-class quadratic Growth Mixture Model (GMM) with random intercepts and slopes.

|  |  | **2-class quadratic GMM** | | | | |
| --- | --- | --- | --- | --- | --- | --- |
|  |  | **Class 1** | |  | **Class 2** | |
| Year |  | Estimated | Observed |  | Estimated | Observed |
| 1 |  | 3.60 | 5.16 |  | 10.32 | 9.93 |
| 2 |  | 2.94 | 2.51 |  | 10.28 | 10.03 |
| 3 |  | 2.35 | 1.69 |  | 10.50 | 10.16 |
| 4 |  | 1.84 | 1.49 |  | 10.59 | 10.32 |
| 5 |  | 1.41 | 1.41 |  | 10.53 | 10.51 |
| 6 |  | 1.06 | 1.39 |  | 10.78 | 10.73 |
| 7 |  | 0.79 | 1.43 |  | 11.05 | 10.97 |
| 8 |  | 0.59 | 0.82 |  | 11.69 | 11.25 |
| 9 |  | 0.47 | 0.57 |  | 11.39 | 11.55 |
| 10 |  | 0.43 | 0.41 |  | 11.82 | 11.88 |

**Supplementary Table 6.** Estimated and observed means of four-class quadratic Growth Mixture Model (GMM) with random intercepts and slopes.

|  |  | **4-class quadratic GMM^a^** | | |  | |  |  | |  |  | |
| --- | --- | --- | --- | --- | --- | --- | --- | --- | --- | --- | --- | --- |
|  |  | **Class 1** | |  | **Class 2** | |  | **Class 3** | |  | **Class 4** | |
| Year |  | Estimated | Observed |  | Estimated | Observed |  | Estimated | Observed |  | Estimated | Observed |
| 1 |  | 3.04 | 4.66 |  | 6.26 | 6.22 |  | 10.72 | 11.34 |  | 10.53 | 10.75 |
| 2 |  | 2.25 | 1.88 |  | 4.86 | 4.07 |  | 11.14 | 10.55 |  | 10.87 | 10.88 |
| 3 |  | 1.58 | 0.99 |  | 3.96 | 3.78 |  | 11.15 | 10.47 |  | 11.16 | 11.15 |
| 4 |  | 1.04 | 0.78 |  | 3.54 | 4.20 |  | 10.77 | 10.17 |  | 11.41 | 11.41 |
| 5 |  | 0.63 | 0.43 |  | 3.61 | 2.87 |  | 9.98 | 11.08 |  | 11.62 | 11.60 |
| 6 |  | 0.34 | 0.59 |  | 4.17 | 3.36 |  | 8.80 | 9.52 |  | 11.78 | 11.91 |
| 7 |  | 0.19 | 0.82 |  | 5.22 | 4.35 |  | 7.22 | 7.48 |  | 11.90 | 12.00 |
| 8 |  | 0.16 | 0.44 |  | 6.76 | 9.69 |  | 5.25 | 4.33 |  | 11.97 | 11.95 |
| 9 |  | 0.26 | 0.44 |  | 8.78 | 8.53 |  | 2.87 | 1.73 |  | 12.00 | 11.82 |
| 10 |  | 0.48 | 0.41 |  | 11.30 | 10.91 |  | 0.09 | 0.44 |  | 11.99 | 12.00 |

**^a^** Average latent class probabilities for most likely latent class membership:

Class 1 Class 2 Class 3 Class 4

0.991 0.996 1.00 0.986

**Supplementary Table 7.** Baseline socio-demographic and clinical characteristics by latent trajectories, descriptive data.

|  | Remitting  row n (row %) | | Late decline  row n (row %) | | Late improvement  row n (row %) | | Persistent  row n (row %) | |
| --- | --- | --- | --- | --- | --- | --- | --- | --- |
| Study centre  London  Nottingham | **119**  **73** | **(54.8)**  **(67.0)** | 11  6 | (5.1)  (5.5) | **12**  **4** | **(5.5)**  **(3.7)** | **75**  **26** | **(34.6)**  **(23.9)** |
| Sex  Men  Women | 98  94 | (53.9)  (65.3) | **8**  **9** | **(4.4)**  **(6.3)** | **11**  **5** | **(6.0)**  **(3.5)** | **65**  **36** | **(35.7)**  **(25.0)** |
| Age  Mean  SD | 29.7  10.0 | | 29.2  7.2 | | 28.3  8.3 | | 30.6  10.3 | |
| Ethnicity  White British  Other White  Black Caribbean  Black African  Asian  Other | **80**  12  **47**  26  13  14 | **(65.6)**  (52.2)  **(46.5)**  (61.9)  (65.0)  (77.8) | **4**  2  **7**  3  0  1 | **(3.3)**  (8.7)  **(6.9)**  (7.1)  -  (5.6) | 7  1  6  2  0  0 | (5.7)  (4.4)  (5.9)  (4.8)  -  - | **31**  8  **41**  11  7  3 | **(25.4)**  (34.8)  **(40.6)**  (26.2)  (35.0)  (16.7) |
| Education^  University  Further  GCSE  School | 29  50  51  54 | (15.8)  (27.2)  (27.7)  (29.4) | 1  6  5  4 | (6.3)  (37.5)  (31.3)  (25.0) | 1  4  2  9 | (6.3)  (25.0)  (12.5)  (56.3) | 4  31  26  36 | (4.1)  (32.0)  (26.8)  (37.1) |
| Social disadvantage*  0, 1  2  3  4 | **44**  42  41  **28** | **(75.9)**  (60.0)  (58.8)  **(45.9)** | 2  1  5  5 | (3.5)  (1.4)  (7.0)  (8.2) | 2  3  2  6 | (3.5)  (4.3)  (2.8)  (9.8) | **10**  24  23  **22** | **(17.2)**  (34.3)  (32.4)  **(36.1)** |
| Substance Use†  Non-problematic use  Abuse  Dependence | 100  36  40 | (56.8)  (20.5)  (22.7) | 12  2  1 | (80.0)  (13.3)  (6.7) | 4  3  9 | (25.0)  (18.8)  (56.3) | 48  25  20 | (51.6)  (26.9)  (21.5) |
| Diagnosis  Non-affective  Affective | 121  71 | (50.6)  (81.6) | 15  **2** | (6.3)  **(2.3)** | 11  5 | (4.6)  (5.8) | 92  9 | (38.4)  (10.3) |
| DUP (weeks)**  Median  IQR | **5.9**  **2-25** | | **9.9**  **1-21** | | **4.3**  **3-22** | | **17.4**  **5-87** | |
| Premorbid IQ (quartiles)‡  1st (highest)  2nd  3rd  4th (lowest) | 27  19  19  16 | (33.3)  (23.5)  (23.5)  (19.8) | 0  2  2  2 | (0.0)  (33.3)  (33.3)  (33.3) | 1  2  2  4 | (11.1)  (22.2)  (22.2)  (44.4) | 4  10  15  12 | (9.8)  (24.4)  (36.6)  (29.3) |

Note: Class 1: Remitting: course characterised by remitting periods of symptoms, which became shorter and less frequent over time; Class 2: Late decline: course characterised, initially, by remitting periods of symptoms, with more persistent symptoms over time; Class 3: Late improvement: course characterised, initially, by persistent symptoms, with remitting periods of symptoms later; Class 4: Persistent: a course characterised by persistent or long periods of symptoms throughout.

^ 13 missing

* 62 missing

† 26 missing

** 24 missing

‡ 189 missing

**Supplementary Table 8.** Latent trajectories and treatment resistence.*

| Trajectories | Responder  n (%) | | Never adequate treatment  n (%) | | Treatment resistant, at onset  n (%) | | Treatment resistant, late  n (%) | |
| --- | --- | --- | --- | --- | --- | --- | --- | --- |
| 1 Remitting | 130 | (86.1) | 6 | (85.7) | 6 | (13.0) | 5 | (45.5) |
| 2 Late decline | 6 | (4.0) | 0 | (0.0) | 1 | (2.2) | 4 | (36.4) |
| 3 Late improve | 12 | (8.0) | 0 | (0.0) | 3 | (6.5) | 0 | (0.0) |
| 4 Continuous | 3 | (2.0) | 1 | (14.3) | 36 | (78.3) | 2 | (18.2) |

Note 1: Class 1: Remitting: course characterised by remitting periods of symptoms, which became shorter and less frequent over time; Class 2: Late decline: course characterised, initially, by remitting periods of symptoms, with more persistent symptoms over time; Class 3: Late improvement: course characterised, initially, by persistent symptoms, with remitting periods of symptoms later; Class 4: Persistent: a course characterised by persistent or long periods of symptoms throughout.

Note 2: Given that many cells have 0 or a small number of observations, and patterns of overlap between trajectories and other course and outcome variables are clear, test statistics were not calculated.

* missing, n 111


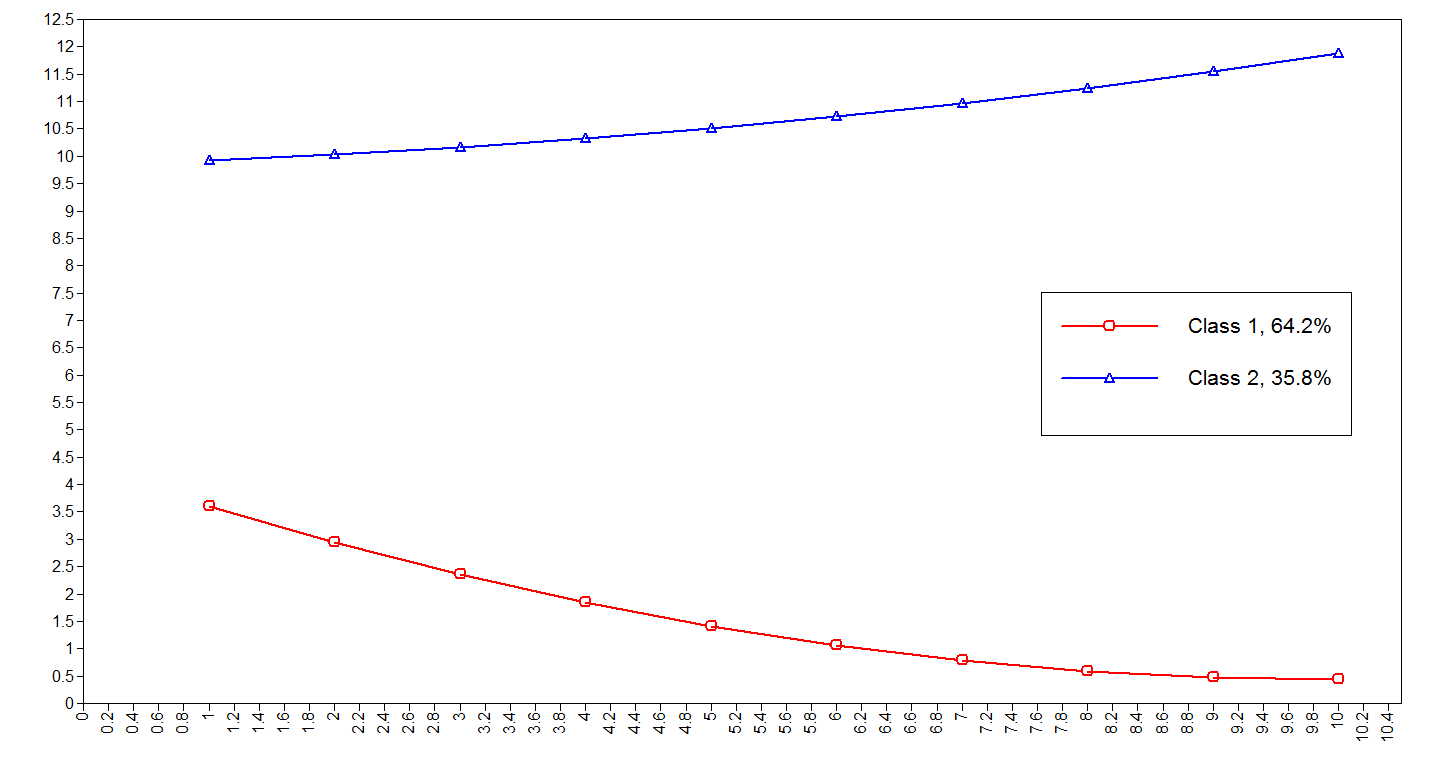


year of follow-up

mean number of months symptomatic

**Supplementary Figure 1.** Estimated latent trajectories of 2-class quadratic GMM (Model 2.2.2, see Table 1) for number of months psychotic per year (n=326)

Trajectory 1


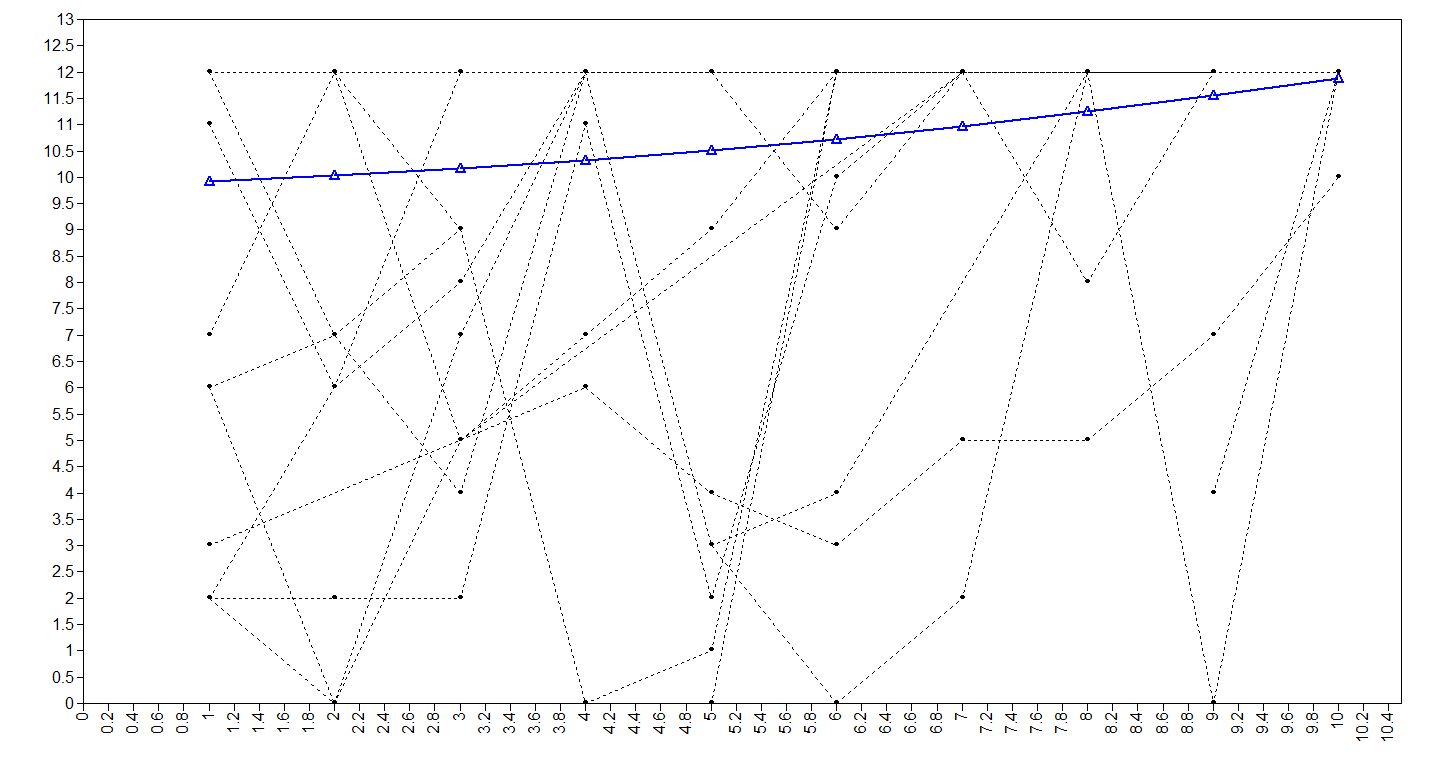


year of follow-up

mean number of months symptomatic

Trajectory 2


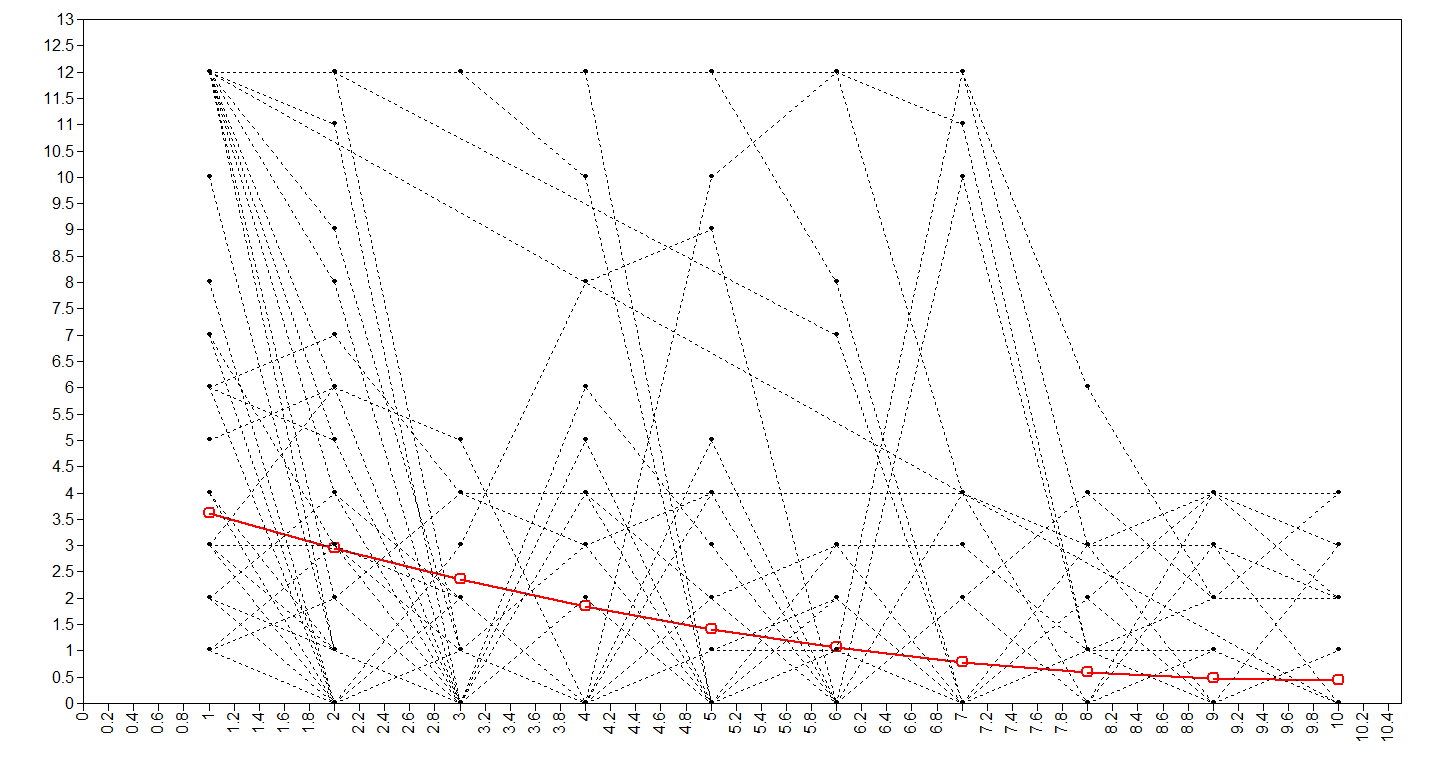


year of follow-up

mean number of months symptomatic

**Supplementary Figure 2.** Estimated means and observed values of 2-class quadratic GMM in randomly selected 100 subjects (Model 2.2.2, see Table 1) for number of months psychotic per year (n=326)
